# Improving *Escherichia coli* membrane integrity and fatty acid production by expression tuning of FadL and OmpF

**DOI:** 10.1186/s12934-017-0650-8

**Published:** 2017-02-28

**Authors:** Zaigao Tan, William Black, Jong Moon Yoon, Jacqueline V. Shanks, Laura R. Jarboe

**Affiliations:** 1 4134 Biorenewables Research Laboratory, Department of Chemical and Biological Engineering, Iowa State University, Ames, IA 50011 USA; 2 Department of Chemical Engineering and Materials Sciences, University of California, 916 Engineering Tower Irvine, Irvine, CA 92697-2575 USA

**Keywords:** Membrane engineering, Membrane integrity, Outer membrane protein, Tolerance, Fatty acid production

## Abstract

**Background:**

Construction of microbial biocatalysts for the production of biorenewables at economically viable yields and titers is frequently hampered by product toxicity. Membrane damage is often deemed as the principal mechanism of this toxicity, particularly in regards to decreased membrane integrity. Previous studies have attempted to engineer the membrane with the goal of increasing membrane integrity. However, most of these works focused on engineering of phospholipids and efforts to identify membrane proteins that can be targeted to improve fatty acid production have been unsuccessful.

**Results:**

Here we show that deletion of outer membrane protein *ompF* significantly increased membrane integrity, fatty acid tolerance and fatty acid production, possibly due to prevention of re-entry of short chain fatty acids. In contrast, deletion of *fadL* resulted in significantly decreased membrane integrity and fatty acid production. Consistently, increased expression of *fadL* remarkably increased membrane integrity and fatty acid tolerance while also increasing the final fatty acid titer. This 34% increase in the final fatty acid titer was possibly due to increased membrane lipid biosynthesis. Tuning of *fadL* expression showed that there is a positive relationship between *fadL* abundance and fatty acid production. Combinatorial deletion of *ompF* and increased expression of *fadL* were found to have an additive role in increasing membrane integrity, and was associated with a 53% increase the fatty acid titer, to 2.3 g/L.

**Conclusions:**

These results emphasize the importance of membrane proteins for maintaining membrane integrity and production of biorenewables, such as fatty acids, which expands the targets for membrane engineering.

**Electronic supplementary material:**

The online version of this article (doi:10.1186/s12934-017-0650-8) contains supplementary material, which is available to authorized users.

## Background

Construction of microbial cell factories for production of biorenewable fuels and chemicals is a promising alternative to current petroleum-driven industries [1, 2]. A variety of microorganisms have been engineered for production of bulk chemicals, biofuels and high-value, fine chemicals [3–7]. However, performance of some biocatalysts can be restricted by various detrimental effects, including toxicity of the product or components of the feedstock [8, 9]. A variety of adverse effects could be the cause of this toxicity, e.g. intracellular acidification; DNA, RNA, protein and membrane damage [10]. Among these, membrane damage has been recognized as a common problem [11–15].

Membrane damage can be compared to a reaction vessel that is vulnerable to corrosion by its contents. In this scenario, a typical response would be to change the composition of the reaction vessel in order to increase resistance to corrosion. For microbial biocatalysts, the composition, function and physical properties of the membrane can be altered through targeted, rational genetic manipulation. Such genetic manipulation is consistent with Cameron and Tong’s fifth application of cellular and metabolic engineering, “modification of cell properties” [16]. When enzymes, transporters and regulators are involved in this membrane engineering, it is also consistent with Bailey’s 1991 definition of metabolic engineering as “the improvement of cellular activities by manipulation of enzymatic, transport, and regulatory function of the cell with the use of recombinant DNA technology” [17].

This work focuses on membrane engineering to improve production of fatty acids, an attractive class of biorenewable chemicals which can be catalyzed to a variety of products with a large potential market, e.g. alkanes, olefins, esters, fatty aldehydes, and fatty alcohols [18–22]. Unfortunately, these fatty acids have been reported to cause a decrease in membrane integrity of *E. coli* during both exogenous challenge and endogenous production [14]. Engineering of membrane phospholipids has proven as a powerful tool in addressing membrane integrity. Decreasing incorporation of medium-chain fatty acids into the membrane increased the average membrane lipid length, decreased the toxicity of fatty acids and increased fatty acid (C12–C14) production in rich medium from 0.60 to 1.36 g/L [23]. Expression of a thioesterase from *Geobacillus* sp. Y412MC10 that prevents medium-chain unsaturated acyl-ACPs from being incorporated into the phospholipids was shown to increase membrane integrity during fatty acid production, but there was no increase in fatty acid (C8–C14) production after 24 h in rich medium, with titers of 0.65 g/L observed with and without expression of the secondary thioesterase [24]. Both of these works demonstrate the feasibility of engineering the membrane lipid composition in order to increase membrane integrity and possibly enhance fatty acid tolerance and production [23, 24].

As efforts continue to increase the membrane integrity during production of membrane-damaging compounds, it becomes increasingly important to provide a sufficient route of product export. Several studies have shown that increasing the expression of transporters can increase production of inhibitory compounds, such as valine [25] and limonene [8]. With the goal of using this strategy to improve fatty acid production, sixteen possible fatty acid transporters were characterized for their role in fatty acid tolerance and production [26]. This previous study identified several transporters that increased fatty acid tolerance when their expression was increased, but did not identify any such transporters that increased fatty acid production.

The transporters OmpF and FadL were part of the previous study. The OmpF protein exists as a trimer in the outer membrane and participates in the transport of sugars, ions, antibiotics and proteins across the outer membrane [27, 28]. FadL is an outer membrane ligand gated channel that functions in the uptake of exogenous long-chain fatty acids (LCFA), [29, 30], especially palmitic acid (C16:0) and oleic acid (C18:1), yet shows no binding to short-chain fatty acids (SCFA, <C10) [31]. Even though the previous characterization observed that deletion of *ompF* and *fadL* had no impact on fatty acid production [26], several other reports related to these two transporters (Table 1) motivated the further exploration of their role in fatty acid tolerance and production described here.

**Table 1:** Previous reports of the role of OmpF and FadL in tolerance of membrane-damaging compounds

Compound	Condition	Result	Reference
OmpF, outer membrane porin F			
C_8_–C_14_ fatty acids	Production of ~1 g/L fatty acids during growth in LB with glycerol, 37 °C	Deletion of *ompF* from a derivative of MG1655 had no impact on cell viability or membrane integrity	[26]
Octanoic acid (C8)	Challenge with up to 20 mM C8 in minimal media with glucose, tryptone and yeast extract at pH 7.0 and 37 °C	Deletion of *ompF* from BW25113 decreased sensitivity to C8, and increased expression of *ompF* increased sensitivity to C8. Sensitivity was assessed via the maximum OD. Deletion of *ompF* decreased the magnitude of intracellular acidification	[32]
Phenylpropanoids	Challenge with 1 g/L rutin, naringenin or resveratrol in M9 medium with casamino acids and glucose at 30 °C	Increased expression of *ompF* in BL21 increased the maximum specific growth rate during challenge. Decreased growth rate during challenge was observed when *ompF* expression was decreased	[33]
FadL, long-chain fatty acid outer membrane porin			
C_8_–C_14_ fatty acids	Production of ~1 g/L fatty acids during growth in LB with glycerol at 37 °C	Deletion of *fadL* from a derivative of MG1655 had no impact on cell viability or membrane integrity	[26]
Palmitic and ω-hydroxy palmitic acids	Addition of 1 mM palmitic acid in potassium phosphate buffer with glucose or glycerol, 30 °C	Increased expression of *fadL* increased conversion of palmitic acid to ω-hydroxy palmitic acid. The increase was smaller in the presence of glycerol than glucose	[34]
Phenol	Challenge with phenol at 50–75% of the MIC in LB at 37 °C	Deletion of *fadL* from BW25113 had no impact on survival	[37]
Octane	Addition of ~20 vol% octane in LB at 37 °C	Deletion of *fadL* from a BW25113 derivative abolished conversion of octane to octanol, octanal and octanoic acid	[35]
Hexane	Challenge with 10 vol% hexane in LB at 37 °C	Deletion of *fadL* from BW25113 increased survival, as assessed by OD	[35]
Phenylpropanoids	Challenge with 1 g/L rutin, naringenin or resveratrol in minimal medium with casamino acids and glucose at 30 °C	Increased expression of *fadL* in BL21 increased the maximum specific growth rate during challenge. Decreased growth rate during challenge was observed when *fadL* expression was decreased	[33]

Two 2015 publications directly implicated OmpF in tolerance of exogenously supplied inhibitors, though in one case OmpF played a protective role and in the other it played a damaging role. Most relevant to our goal of improving fatty acid production is the demonstration that deletion of *ompF* dampened octanoic acid toxicity, with evidence that this deletion of *ompF* reduced SCFA entry into cells [32] (Table 1). This reduced entry of SCFA into cells was assessed by measuring the decrease in intracellular pH during challenge with exogenously supplied octanoic acid. Contrastingly, OmpF was found to be directly related to tolerance of three exogenously provided phenylpropanoids: rutin, naringenin and resveratrol [33]. Specifically, strains with increased expression of OmpF showed increased tolerance to these compounds and strains with decreased expression of OmpF showed decreased tolerance, leading to the proposition that OmpF participates in the removal of phenylpropanoids from the cell interior. Thus, OmpF showed a negative role in SCFA tolerance and a positive role in phenylpropanoid tolerance.

There are also reports of FadL being involved in fatty acid production and tolerance to some inhibitors (Table 1). Increased expression of *fadL* resulted in increased conversion of exogenously supplied palmitic acid to ω-hydroxy palmitic acid [34]. This improved organism performance was attributed to increased uptake of palmitic acid, as data indicated that FadL was not involved in export of the hydroxylated product. Similarly, FadL seemed to play a crucial role in the import of octane for production of octanol, octanal and octanoic acid [35]. Specifically, production of these compounds from exogenously supplied octane was abolished when *fadL* was deleted. However, it was noted that this deletion of *fadL* increased survival during challenge with hexane, with the conclusion that FadL was the main route of hexane entry into the cell [35]. The phenylpropanoid studies described above also noted that FadL abundance was directly related to tolerance of exogenously supplied rutin, naringenin and resveratrol, the same trend was observed for OmpF, with the interpretation that FadL was involved in repairing membrane damage caused by these compounds [33]. However, even though phenol toxicity is often attributed to membrane damage [36], deletion of *fadL* had no impact on survival during phenol challenge [37]. Thus, FadL appears to be important to the uptake of some fatty acids and alkanes, provides protection from the inhibitory effects of phenylpropanoids, provides entry to some harmful alkanes and yet possibly plays no role in repairing the membrane damage caused by phenol.

Here we have taken another look at the role of OmpF and FadL in fatty acid tolerance and production, with the conclusion that OmpF and FadL have opposite effects. Specifically, fatty acid tolerance, fatty acid production and membrane integrity were all increased when *ompF* was deleted or when expression of *fadL* was increased. Concurrent utilization of these two engineering strategies enabled a roughly 50% increase in production of fatty acids (primarily C14, C16:1 and C16), resulting in a final titer of 2.3 g/L. Although we employed a thioesterase specific for LCFA (C14–C16), some SCFAs (e.g. C8 and C10) were also produced. We propose that deletion of *ompF* prevents re-entry of the SCFA and their corresponding toxic effects. Contrastingly, it seems that FadL may enable the recapture of some of the LCFA for use in membrane biosynthesis and repair.

## Methods

### Strains and plasmids

All plasmids and strains used in this study are listed in Table 2. One-step recombination method (FLP-FRT) was employed to perform genetic modifications [38]. *E. coli* K-12 MG1655 was employed as the host strain. For modulating expression of *fadL*, the FRT-*cat*-FRT selection marker linked with four different promoters (M1-12, M1-37, M1-46, M1-93) [6, 39, 40] with varying strengths was employed to regulate expression of the original *fadL* gene, yielding engineered strains M1-12-*fadL*, M1-37-*fadL*, M1-46-*fadL* and M1-93-*fadL*, respectively.

**Table 2:** Strains and plasmids used in this study

Strains/plasmids	Genetic characteristics	Source
Strains		
MG1655	Wild type *E. coli* K-12 strain	Lab collection
Δ*ompF*	MG1655, Δ*ompF*	This study
Δ*fadD*	MG1655, Δ*fadD*	This study
Δ*fadL*	MG1655, Δ*fadL*	This study
Pla-empty	MG1655, pACYC184-Kan	This study
Pla-*fadL*	MG1655, pACYC184-Kan-*fadL*	This study
Gen-empty	MG1655, *ldhA*::FRT-*cat*-FRT	This study
Gen-*fadL*	MG1655, *ldhA*::FRT-*cat*-FRT, *fadL*	This study
M1-12-*fadL*	MG1655, FRT-*cat*-FRT, M1-12-*fadL*	This study
M1-37-*fadL*	MG1655, FRT-*cat*-FRT, M1-37-*fadL*	This study
M1-46-*fadL*	MG1655, FRT-*cat*-FRT, M1-46-*fadL*	This study
M1-93-*fadL*	MG1655, FRT-*cat*-FRT, M1-93-*fadL*	This study
Δ*ompF* + Pla-empty	MG1655, Δ*ompF*, pACYC184-Kan	This study
Δ*ompF* + Pla-*fadL*	MG1655, Δ*ompF*, pACYC184-Kan-*fadL*	This study
Plasmids		
pACYC184-Kan	p15A, pACYC184, Kan^r^	This study
pACYC184-Kan-*fadL*	pACYC184-Kan harboring *fadL*, Kan^r^	This study
pXZ18Z (TE)	pTrc99a-*Ricinus communis* thioesterase-*fabZ*, Amp^r^	[42]

For increasing expression of *fadL*, two different strategies were employed. First, the low-copy plasmid pACYC184-Kan-*fadL*, which harbors the native promoter, open reading frame (ORF), and terminator of *fadL* was transformed to MG1655, resulting in Pla-*fadL*. MG1655 with empty pACYC184-Kan served as the corresponding control (Pla-empty). Second, for increased expression of *fadL* from the chromosome, a second copy of the *fadL* gene was inserted into the MG1655 genome at the *ldhA* site, resulting in Gen-*fadL*. The *ldhA* gene was also deleted from MG1655 to generate strain Gen-empty, which serves as a control for strain Gen-*fadL*. Selection of *ldhA* as the integration site was motivated by previous reports [41].

The pXZ18Z plasmid [42] harboring a thioesterase from *Ricinus communis* and the *E. coli* 3-hydroxyacyl-ACP dehydratase (*fabZ*) was used for long-chain fatty acid (LCFA) production. When necessary, ampicillin, kanamycin and chloramphenicol were used at final concentrations of 100, 50 and 34 mg/L, respectively.

### Strain tolerance characterization

Octanoic acid tolerance was characterized in 50 mL MOPS defined minimal medium with 2.0% (wt/v) dextrose and 10 mM octanoic acid (1.44 g/L) in 250 mL baffled flasks at 220 rpm and initial pH at 7.0, 30 °C. MOPS media contains the following: 8.37 g/L 3-(*N*-morpholino)propanesulfonic acid (MOPS), 0.72 g/L tricine, 2.92 g/L NaCl, 0.51 g/L NH_4_Cl, 1.6 g/L KOH, 50 mg/L MgCl_2_, 48 mg/L K_2_SO_4_, 348 mg/L K_2_HPO_4_, 0.215 mg/L Na_2_SeO_3_, 0.303 mg/L Na_2_MoO_4_·2H_2_O, 0.17 mg/L ZnCl_2_, 2.5 μg/L FeCl_2_·4H_2_O, 0.092 μg/L CaCl_2_·2H_2_O, 0.031 μg/L H_3_BO_3_, 0.020 μg/L MnCl_2_·4H_2_O, 0.0090 μg/L CoCl_2_·4H_2_O, and 0.0020 μg/L CuCl_2_·4H_2_O [43, 44]. Specific growth rate μ (h^−1^) was calculated by fitting the equation OD = OD_0_e^μt^ over the duration of the exponential growth phase. OD was measured at 550 nm and all estimated μ values had an R^2^ of at least 0.95 [45]. Dry cell weight (DCW) was calculated from the optical density at 550 nm (1 OD_550_ = 0.333 g DCW/L).

### Membrane integrity characterization

Cells were centrifuged, washed twice, and then resuspended in PBS buffer (pH 7.0) at a final OD_550_ of ~1. One hundred microliter (100 μL) of this suspension was mixed with 900 μL of PBS buffer and SYTOX Green (Invitrogen) was added to a final concentration of 5.0 μM. After resting at room temperature for 15 min, cells were analyzed by a BD Biosciences FACSCanto II flow cytometer equipped with standard factory-installed 488 nm excitation laser, signal collection optics, and fluorescence emission filter configuration. Instrument sheath fluid was filtered (0.22 μm) PBS buffer. Green fluorescence from stained cells was collected in the FL1 channel (525/50 nm). Forward scatter (FSC), side scatter (SSC), and FL1 (Green) parameters were collected as logarithmic signals. All data collections were performed at low flow rate setting (~12 μL/min) and cell concentrations were such that the event rate was below 5000 events/s. All samples were analyzed immediately after staining. Background noise and small debris was eliminated from data collection via a side scatter signal threshold that was established by examining samples containing only SYTOX Green staining buffer. Bacteria in SYTOX Green-stained samples were readily identified on the basis of FSC and SSC signals and an appropriate “Cell” gate was drawn to limit FL1 analysis to bacteria and exclude non-cell events. A minimum of 20,000 cell-gated events were collected for each sample. Green fluorescence data for these “cell” events were plotted as histograms showing the signal distribution of bacteria in the sample [14]. Flow cytometry data for this work is available via Flow Repository (https://flowrepository.org) FR-FCM-ZY2B.

### Membrane lipid composition characterization

The membrane lipids were extracted by using the Bligh and Dyer method with minor modifications [14, 46]. Cells were centrifuged, washed twice with cold double-distilled water (ddH_2_O), resuspended in 1.4 mL methanol and transferred to a new glass tube. Ten μicroliter of 1 μg/μL pentadecanoic acid (C15:0) dissolved in ethanol was added as internal standard. Then, samples were sonicated, incubated at 70 °C for 15 min and centrifuged at 5000×*g* for 5 min. The supernatant was transferred to a new glass tube and the cell pellet was resuspended in 0.75 mL of chloroform, shaken at 37 °C, 150 rpm for 5 min. Transferred supernatant and pellet suspension were combined, vortexed for 1 min and centrifuged at 5000×*g* for 2 min. The bottom phase was transferred to a new glass tube and dried under nitrogen gas. Two milliliter of methanol:sulfuric acid (98:2 v/v) mixture was added and the mixture was vortexed and incubated at 80 °C for 30 min. Finally, 2 mL of 0.9% (wt/v) sodium chloride (NaCl) and 1 mL of hexane were added, vortexed and centrifuged at 2000×*g* for 2 min. The top hexane layer was then analyzed by gas chromatography–mass spectrometry (GC–MS). The temperature for GC–MS analysis was initially held at 50 °C for 2 min, ramped to 200 °C at 25 °C/min, held for 1 min, then raised to 315 °C at 25 °C/min, held for 2 min. Helium was used as a carrier gas and the flow rate was 1 mL/min through a DB-5MS separation column (30 m, 0.25 mm ID, 0.25 μm, Agilent). Methods for calculating average membrane lipid length and lipid saturated:unsaturated ratio can be found in [14].

### Membrane lipid content measurement

Thirty milliliters of mid-log *E. coli* cells were centrifuged, washed by ddH_2_O and adjusted to OD_550_ ~10. Then, 1.8 mL of cell suspension was centrifuged at 14,000×*g* for 5 min and the resulting cell pellets were resuspended in 1.4 mL methanol. As described in “Membrane lipid composition characterization” section, the total membrane bound fatty acid was measured. Given that membrane-bound fatty acids account for 71% (w/w) of lipid mass [47], we use the following formula to calculate the membrane lipid content: total membrane lipid (mg/g DCW) = membrane fatty acids (mg)/0.71 × g DCW.

### Real-time quantitative PCR

Bacterial cultures were grown and collected by centrifugation at 10,000×*g* for 2 min. Total RNA was extracted by using the RNeasy Mini Kit (Qiagen), and the residual DNA was removed by TURBO DNA-free™ Kit (Life Technology). Superscript III First-Strand Synthesis SuperMix (Invitrogen) was employed for the cDNA synthesis, then the cDNA was diluted 100-fold and used as template for quantitative real-time PCR (qRT-PCR) analysis with SYBR Green ER™ qPCR SuperMix (Invitrogen). The *E. coli* 16S *rrsA* gene was employed as the housekeeping gene for *fadL* mRNA abundance analysis. Sequences of *fadL* primers for qRT-PCR are CTGAAATGTGGGAAGTGTC/GAAGGTCCAGTTATCATCGT, Primers for *rrsA* are TGGCTCAGATTGAACGC/ATCCGATGGCAAGAGGC. The qRT-PCR was performed with the StepOnePlus™ Real-Time PCR System (Thermo Fisher Scientific). The PCR mixture was held at 95 °C for 10 min and then subjected to 40 cycles of incubation at 95 °C for 15 s, then 60 °C for 1 min.

### Fermentation for fatty acid production

Individual colonies were selected from Luria Broth (LB) plates with ampicillin and inoculated into 3 mL of LB liquid medium with ampicillin for 4 h. Then, 0.5 mL of culture was added to 20 mL LB with ampicillin at 30 °C, 220 rpm overnight for seed culture preparation. Seed culture was collected, resuspended in MOPS 2.0% (wt/v) dextrose medium, and transferred into 50 mL MOPS 2.0% (wt/v) dextrose containing ampicillin and 1 mM of isopropyl-β-d-thiogalactopyranoside (IPTG) in 250 mL baffled flasks. The target initial cell density was OD_550_ ~0.1. Cultures were grown in 250 mL baffled flasks with initial pH 7.0 at 30 °C, 220 rpm for 72 h.

### Determination of carboxylic acid titers

Carboxylic acid production was quantified by an Agilent 7890 gas chromatograph equipped with an Agilent 5975 mass spectroscope using flame ionization detector and mass spectrometer (GC–MS) after carboxylic acid extraction. Briefly, 100 μL of whole liquid media sample was taken and 10 μL of 1 μg/μL C7:0/C11:0/C17:0 was added as internal standards. Two milliliter of ethanol: sulfuric acid (98:2 v/v) mixture was added, mixed and incubated at 65 °C for 30 min. Then, 2 mL of 0.9% (wt/v) NaCl solution and 1 mL of hexane were added, vortexed and centrifuged at 2000×*g* for 2 min. The top hexane layer was then analyzed by GC–MS, as described in “Strain tolerance characterization” section.

### Statistical analysis

The two-tailed t test method was employed to analyze the statistical significance of all data in this study and P value <0.05 is deemed statistically significant.

## Results

### Effects of *ompF* or *fadL* deletion on tolerance and production of fatty acids

**Fig. 1:**
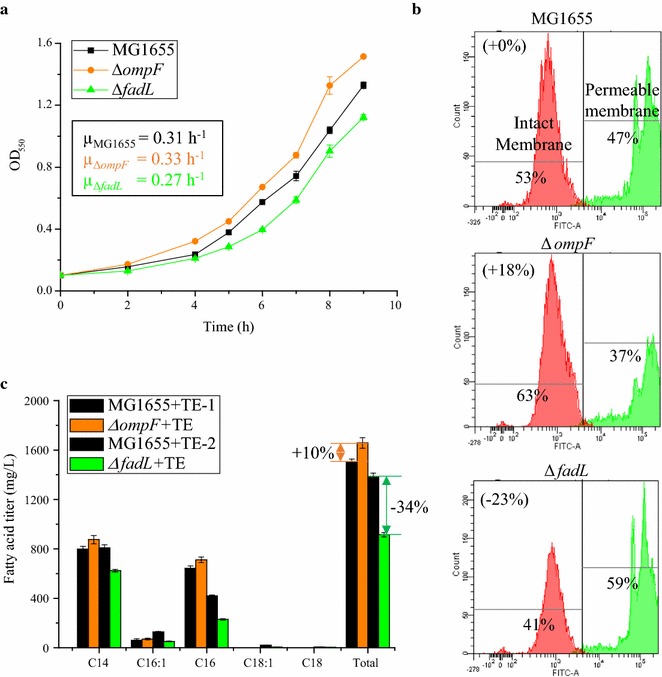
Effects of *ompF* or *fadL* deletion on membrane integrity during short-chain fatty acid challenge, short-chain fatty acid tolerance and production of C12 and C14 fatty acids. **a** Deletion of *ompF* or *fadL* impact the specific growth rate relative to the wild type MG1655 during challenge with 10 mM C8. *Inset values* are the specific growth rate, h^−1^. **b** Deletion of *ompF* or *fadL* alters the percentage of cells with intact membranes (membrane integrity), assessed using SYTOX Green, during challenge with 10 mM C8. **c** Deletion of *ompF* increased fatty acid production and deletion of *fadL* decreased fatty acid production. MG1655 + TE-1 and MG1655 + TE-2 indicates experiments performed with the same strain, but on different days. For **a** and **b**, experiments were performed in MOPS + 2% (wt/v) dextrose shake flasks at 220 rpm 30 °C with an initial pH of 7.0, 10 mM octanoic acid (C8). For **c**, strains carry the pXZ18Z plasmid (TE) for LCFA (C14–C16) production. Fermentations were performed in MOPS + 2% (wt/v) dextrose shake flasks at 220 rpm 30 °C with an initial pH of 7.0, 1.0 mM IPTG. Values are the average of at least three biological replicates with *error bars* indicating one standard deviation. Percent increase values are shown only for differences that were deemed statistically significant (P < 0.05)

It was previously reported that OmpF facilitates transport of SCFA, such as octanoic acid (C8), into *E. coli*, and that deletion of *ompF* in *E. coli* BW25113 decreased the impact of C8 on biomass production [32]. To evaluate the effect of OmpF on C8 tolerance in MG1655, we also constructed an *ompF* deletion strain (Δ*ompF*) and confirmed that this engineering strategy improved tolerance to C8. In the absence of C8, the specific growth rates (µ) of both strains were approximately 0.39 h^−1^. During C8 challenge, the specific growth rate of the Δ*ompF* mutant was 0.33 h^−1^, which is 7% higher than that of MG1655 (0.31 h^−1^) (Fig. 1a), which is consistent with the previous report [32].

Decreased membrane integrity has been previously described as a primary cause of C8 toxicity, where decreased membrane integrity is evidenced by leakage of metabolites and ions, such as Mg^2+^, out of the cell or the entry of membrane-impermeable molecules, such as SYTOX, into the cell [14, 24, 48]. We next characterized the membrane integrity changes after disruption of *ompF*. Consistent with the growth results, deletion of *ompF* dampened the impact of C8 on membrane integrity. Specifically, the percentage of cells with intact membranes, i.e. SYTOX impermeable, during challenge with exogenously provided 10 mM C8, increased by 18% compared with the wild-type control strain (P < 0.05) (Fig. 1b).

Given that increased tolerance might lead to increased production of bio-products, we next applied the *ompF* deletion strategy to fatty acid production. The plasmid pXZ18Z (TE) harboring the heterologous thioesterase from *R. communis* [42], which primarily releases tetradecanoic acid (C14:0), palmitoleic acid (C16:1) and hexadecanoic acid (C16:0), was transformed into the Δ*ompF* strain and the corresponding control for fatty acid production in minimal MOPS 2.0% (wt/v) dextrose medium. We observed that deletion of *ompF* increased fatty acid production (Fig. 1c): in the Δ*ompF* + TE mutant, the titer of C14:0 was increased by 10% (P = 0.03) to 875 mg/L, C16:1 was increased by 17% (P = 0.24) to 71 mg/L and C16:0 was increased by 11% (P = 0.01) to 711 mg/L. All of these increases led to a 10% improvement of total fatty acids produced by the Δ*ompF* + TE mutant compared to MG1655 + TE strain, with titers of 1500 ± 20 and 1660 ± 40 mg/L, respectively (P = 0.005). It should be noted that previous studies concluded that deletion of *ompF* from *E. coli* strain TY05 did not significantly increase fatty acid (C8–C14) production [26]. The difference from this previous report and the findings presented here may be due to the use of different thioesterases (from *U. californica* vs. from *R. communis*), growth media (nutrient-rich LB + 0.4% (v/v) glycerol vs. minimal MOPS + 2% (wt/v) glucose) and temperature (37 vs. 30 °C).

While OmpF has been previously characterized in terms of SCFA transport, FadL predominantly functions in the uptake of LCFA [29, 30]. To investigate the effect of FadL on fatty acid tolerance and production, a *fadL* deletion mutant (Δ*fadL*) was constructed. Interestingly, the Δ*fadL* mutant showed decreased tolerance to C8. For example, the specific growth rate of the Δ*fadL* strain was 12% lower than that of MG1655 (0.27 vs. 0.31 h^−1^) (P < 0.05) (Fig. 1a). Further membrane characterization showed that the percentage of cells with intact membranes was 23% lower for the Δ*fadL* strain than MG1655 (P < 0.05) (Fig. 1b). When this *fadL* deletion strategy was applied to fatty acid production (+TE), titers of C14:0 decreased by 23% to 623 mg/L, C16:1 decreased by 60% to 51 mg/L and C16:0 decreased by 45% to 230 mg/L. Each of these changes had a P value less than 0.05. Together, these changes led to a 34% reduction of total fatty acids in the Δ*fadL* + TE mutant compared with MG1655 + TE strain (from 1390 ± 30 to 920 ± 20 mg/L) (P < 0.05) (Fig. 1c). It should be noted that the fatty acid titer of MG1655 + TE here (1390 ± 30 mg/L) is slightly lower than the 1500 ± 20 mg/L described above for the *ompF* results, due to differences between batches, similar to the results described elsewhere [26]. As with deletion of *ompF*, our results differ from previous reports of the effect of *fadL* deletion on fatty acid production. This previous characterization employed *E. coli* strain TY05 in rich medium with glycerol and found no significant change in production of C8–C14 fatty acids upon deletion of *fadL* [26]. However, our observation that deletion of *fadL* can increase sensitivity to membrane-damaging short-chain fatty acids is consistent with observations made for phenylpropanoid tolerance [33].

### Increased expression of *fadL* increased fatty acid tolerance and production

**Fig. 2:**
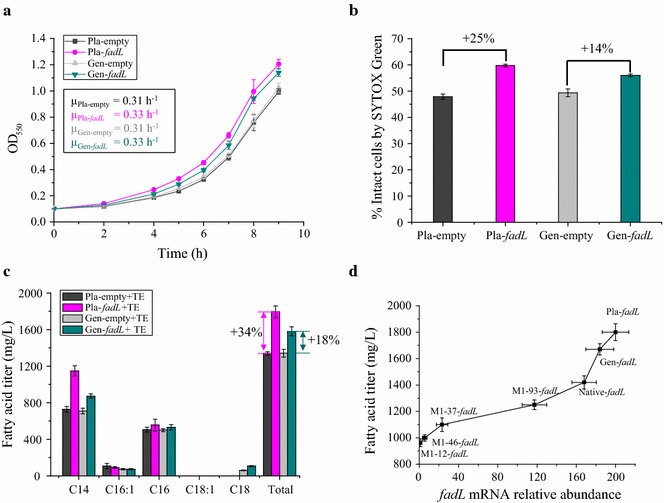
Increased expression of *fadL* increases membrane integrity, fatty acid tolerance and production. **a** Increased expression of *fadL* from a plasmid (Pla-*fadL*) or a genomic insertion (Gen-*fadL*) both increase the specific growth rate relative to the corresponding controls (Pla-empty, Gen-empty) during challenge with 10 mM C8. *Inset values* are the specific growth rate, h^−1^. **b** Percentage of cells with intact membrane (membrane integrity), assessed using SYTOX Green. Strains with increased expression of *fadL*, Pla-*fadL* and Gen-*fadL*, have improved membrane integrity relative to their corresponding controls, Pla-empty and Gen-empty, during challenge with 10 mM C8. **c** Strains with increased expression of *fadL*, Pla-*fadL* and Gen-*fadL*, produce more fatty acid than the corresponding controls, Pla-empty and Gen-empty. **d** The *fadL* mRNA relative abundance at 48 h has a positive relationship with the fatty acids titer after 72 h. Four different promoters (M1-12, M1-37, M1-46 and M1-93) were used to replace the native promoter of *fadL*. The mRNA abundance of *fadL* in M1-12-*fadL* strain was set as 1. The 16S *rrsA* gene was used as normalizing factor. For **a** and **b**, experiments were performed in shake flasks containing MOPS + 2% (wt/v) dextrose with 10 mM octanoic acid (C8) at an initial pH of 7.0, shaken at 220 rpm, and maintained at 30 °C. For **c** and **d**, all strains carry the pXZ18Z plasmid (TE, *fabZ*) for LCFA (C14–C16) production. Fermentations were performed in MOPS + 2% (wt/v) dextrose shake flasks at 220 rpm 30 °C with an initial pH of 7.0, 1.0 mM IPTG. Values are the average of at least three biological replicates with *error bars* indicating one standard deviation. Percent increase values are shown only for differences that were deemed statistically significant (P < 0.05). Pla-empty: MG1655 + pACYC184-Kan; Pla-*fadL*: MG1655 + pACYC184-Kan-*fadL*; Gen-empty: MG1655 *ldhA*::FRT-*cat*-FRT; Gen-*fadL*: MG1655 *ldhA*::FRT-*cat*-FRT, *fadL.* TE: pXZ18Z plasmid

Given that the deletion of *fadL* decreased fatty acid tolerance and production, it is reasonable to expect that increased expression of *fadL* might improve fatty acid tolerance and production. To this end, two different strategies were employed in *E. coli* MG1655 for increased expression of *fadL*: plasmid expression (Pla-*fadL*) and genomic integration of a second copy of *fadL* (Gen-*fadL*). Consistent with our hypothesis, both of these increased expression strategies significantly improved C8 tolerance. Specifically, the specific growth rate of Pla-*fadL* (0.33 h^−1^) and Gen-*fadL* (0.33 h^−1^) were 8 and 7% higher than Pla-empty (0.31 h^−1^) and Gen-empty (0.31 h^−1^) (P < 0.05) (Fig. 2a). Membrane damage, as evidenced by entry of the SYTOX nucleic acid dye into the cell, was decreased in the two strains engineered for increased *fadL* expression. Specifically, Pla-*fadL* showed a 25% increase in membrane integrity and Gen-*fadL* had a 14% increase in membrane integrity (P < 0.05) (Fig. 2b).

Further characterization showed that both of the strains with increased *fadL* expression also had increased fatty acid production capability. This significantly (P < 0.05) increased fatty acid titer was observed for C14:0 and the total fatty acid pool, though the increase was slightly higher for C16:1 and C16:0 in both cases (Fig. 2c). Specifically, the plasmid-based strain produced 1150 mg/L of C14:0, 556 mg/L of C16:0 and 1800 mg/L of total fatty acid, which was 57, 10 and 34% higher than the corresponding control encoding the thioesterase and an empty plasmid. This control strain produced 728 mg/L C14:0, 505 mg/L C16:0 and 1340 mg/L total fatty acids. A similar trend was also observed for genome-based *fadL* expression tuning: 872 mg/L of C14:0, 531 mg/L of C16:0 and 1580 mg/L of total fatty acids were produced by the engineered Gen-*fadL* + TE strain, which was 23, 6 and 18% higher than in the 710, 500 and 1340 mg/L produced by the corresponding Gen-empty + TE control. These results demonstrate the effectiveness of increasing *fadL* expression for increasing fatty acid production.

In order to further characterize the relationship between the expression level of *fadL* and fatty acid production, additional strains were constructed (+TE) and characterized. Specifically, different promoters (M1-12, M1-37, M1-46, M-93) with varied strengths [6, 39, 40] were employed to regulate the expression of the native *fadL* (Fig. 2d). A positive relationship between mRNA relative abundance of *fadL* and fatty acid titers was observed (Fig. 2d). For instance, mRNA relative abundance of *fadL* increased nearly 120-fold in M1-93-*fadL* strain relative to M1-12-*fadL* (of which *fadL* expression level was deemed as 1), and it also produced 1250 mg/L of fatty acid, which is 37% higher than the 915 mg/L produced by M1-12-*fadL*. It should be noted that expression level of *fadL* under all artificial promoters used here is lower than the native promoter, which suggests that expression of *fadL* is held at a relatively high level in *E. coli* MG1655.

### Deletion of *ompF* and increased expression of *fadL* have an additive effect in increasing fatty acid production

Given that deletion of *ompF* and increased expression of *fadL* were each found to increase tolerance and production of fatty acids, we proposed that combinatorial utilization of both engineering strategies would further increase performance. To this end, the plasmid-based expression of *fadL* was selected as the strategy for increasing expression of *fadL*, due to its substantial increase in tolerance and production of fatty acid.

**Fig. 3:**
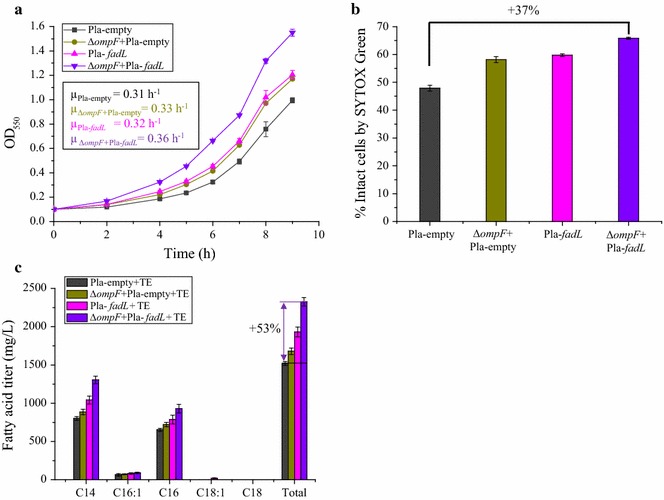
Deletion of *ompF* and increased expression of *fadL* have an additive effect on increasing membrane integrity, fatty acid tolerance and production. **a** Combinatorial deletion of *ompF* (Δ*ompF*) and increased expression of *fadL* (Pla-*fadL*) increases the specific growth rate during challenge with 10 mM C8 relative to the starting strain (Pla-empty), individual *ompF* deletion strain (Δ*ompF* + Pla-empty), and individual overexpression of *fadL* (Pla-*fadL*). *Inset values* are the specific growth rate, h^−1^ **b** Percentage of cells with intact membrane (membrane integrity), assessed using SYTOX Green. Combinatorial deletion of *ompF* and increased expression of *fadL* improves membrane integrity during challenge with 10 mM C8 relative to Pla-empty, Δ*ompF* + Pla-empty and Pla-*fadL* strains. **c** The combined implementation of *ompF* deletion and increased expression of *fadL* supports increased fatty acid titers relative to each engineering strategy implemented individually. For **a** and **b**, experiments were performed in MOPS + 2% (wt/v) dextrose shake flasks at 220 rpm 30 °C with an initial pH of 7.0, 10 mM octanoic acid (C8). For **c**, all strains carry the pXZ18Z plasmid (TE, *fabZ*) for LCFA (C14–C16) production. Fermentations were performed in MOPS + 2% (wt/v) dextrose shake flasks at 220 rpm 30 °C with an initial pH of 7.0, 1.0 mM IPTG. Values are the average of at least three biological replicates with *error bars* indicating one standard deviation. Percent increase values are shown only for differences that were deemed statistically significant (P < 0.05). Pla-empty: MG1655 + pACYC184-Kan; Δ*ompF* + Pla-empty: MG1655, Δ*ompF* + pACYC184-Kan; Pla-*fadL*: MG1655 + pACYC184-Kan-*fadL*; Δ*ompF* + Pla-*fadL*: MG1655, Δ*ompF* + pACYC184-Kan-*fadL*

Consistent with our hypothesis, combinatorial utilization of the *ompF* deletion and increased expression of *fadL* was found to have an additive effect for improving tolerance to C8 (Fig. 3a). The specific growth rate of Δ*ompF* + Pla-*fadL* strain reached up to 0.36 h^−1^ in the presence of 10 mM C8, which exceeds that of Pla-empty (μ = 0.31 h^−1^) by 18%, and is also 10% higher than individual deletion of *ompF* (Δ*ompF* + Pla-empty, μ = 0.33 h^−1^) and 12% higher than individual increased expression of *fadL* (Pla-*fadL*, μ = 0.32 h^−1^) (P < 0.05) (Fig. 3a). Besides increased tolerance, membrane integrity was significantly increased in the Δ*ompF* + Pla-*fadL* strain during challenge with C8. Compared with Pla-empty, the percentage of Δ*ompF* + Pla-*fadL* cells with intact membranes increased by 37% (P < 0.05) (Fig. 3b).

Combination of *ompF* deletion and increased expression of *fadL* also increased the specific growth rate during fatty acid production (data not shown), and final fatty acid titers (Fig. 3c). Specifically, the combination of these engineering strategies in the Δ*ompF* + Pla-*fadL* + TE strain resulted in a specific growth rate of 0.25 h^−1^ in the first 12 h of fermentation, where this exceeds that of Pla-empty (μ = 0.16 h^−1^) by 53% (P < 0.05). Correspondingly, the Δ*ompF* + Pla-*fadL* + TE strain produced 1310 mg/L of C14:0, 90 mg/L of C16:1, 930 mg/L of C16:0 and 2330 mg/L of total fatty acids after 72 h fermentation. These titers are 47, 25, 29 and 38% higher than the strain in which only the *ompF* deletion was implemented (Δ*ompF* + Pla-empty + TE, 885 mg/L of C14:0, 72 mg/L of C16:1, 722 mg/L of C16:0 and 1680 mg/L of total fatty acid) and 25, 10, 18 and 20% higher than the strain in which only the *fadL* overexpression was implemented (Pla-*fadL* + TE, 1040 mg/L of C14:0, 83 mg/L of C16:1, 786 mg/L of C16:0 and 1930 mg/L of total fatty acid). Note that all of these comparisons have P < 0.05, except for C16:1. The combined strain has an approximately 50% improvement in fatty acid titers relative to the corresponding un-engineered control, Pla-empty + TE, which produced 801 mg/L of C14:0, 65 mg/L of C16:1, 653 mg/L of C16:0 and 1520 mg/L of total fatty acid (Fig. 3c). These results again demonstrate the effectiveness of concurrent utilization of *ompF* deletion and increased expression of *fadL* for increasing fatty acid production.

### Functional mechanism of OmpF and FadL on increased membrane integrity

In this study, engineering the abundance of the membrane proteins OmpF and FadL increased membrane integrity, fatty acid tolerance and fatty acid production. Prior studies showed that increasing the average length or the saturated:unsaturated (S/U) ratio of *E. coli* membrane lipids can alleviate the decreased membrane integrity caused by fatty acids [23, 24]. In order to determine whether the increased membrane integrity here could be attributed to such changes in the phospholipid tail distribution, we measured the membrane lipid composition in the wild-type MG1655, Δ*ompF*, Δ*fadL* and Pla-*fadL* strains (Table 3). However, no significant changes in membrane composition were observed. Similarly, the average lipid length in wild-type MG1655 was 16.4 ± 0.2, which is comparable to the value observed for the Δ*ompF*, Δ*fadL* and Pla-*fadL* strains (Table 3). Additionally, the membrane lipid S/U ratio in the wild-type MG1655 was 1.06 ± 0.02, which is similar to the ratios for the Δ*ompF*, Δ*fadL* and Pla-*fadL* strains (Table 3). These results indicate that the previously-described membrane engineering mechanisms of increasing the membrane lipid and S/U ratio are not the underlying reason for increased membrane integrity here.

**Table 3:** Membrane lipid content and composition changes in the wild type MG1655, Δ*ompF*, Δ*fadL*, Pla-*fadL* strains

Strain	Membrane lipid content (mg/g DCW)	Membrane lipid composition (mol %)							Membrane lipid length	Membrane lipid S/U ratio
		C14:0	C16:1	C16:0	C17cyc	C18:1	C18:0	C19cyc		
MG1655	69.4 ± 0.3	1.3 ± 0.1	13.6 ± 0.2	48.5 ± 0.2	14.1 ± 0.1	19.1 ± 0.4	1.70 ± 0.03	1.8 ± 0.1	16.4 ± 0.2	1.06 ± 0.02
Δ*ompF*	71.3 ± 0.5 (+2.7%)	1.1 ± 0.1	13.6 ± 0.1	48.7 ± 0.1	13.6 ± 0.3	19.4 ± 0.1	1.9 ± 0.1	1.7 ± 0.1	16.4 ± 0.1	1.07 ± 0.01
Δ*fadL*	62 ± 3 (−10%)	1.2 ± 0.1	12.7 ± 0.1	48.3 ± 0.4	14.5 ± 0.1	19.4 ± 0.3	1.9 ± 0.1	1.9 ± 0.1	16.4 ± 0.2	1.06 ± 0.01
Pla-*fadL*	78 ± 1 (+13%)	1.3 ± 0.1	13.2 ± 0.2	48.2 ± 0.1	13.4 ± 0.1	20.7 ± 0.1	1.9 ± 0.2	1.2 ± 0.1	16.4 ± 0.1	1.00 ± 0.02

Each value is an average and standard deviation of three biological replicates

All experiments were performed in MOPS + 2% (wt/v) dextrose shake flasks at 220 rpm 30 °C with an initial pH of 7.0, 10 mM octanoic acid (C8). All values are the average of at least three biological replicates with the associated standard deviation indicated. Percent increase values are only shown for differences that were deemed statistically significant (P < 0.05)

*DCW* dry cell weight, *S/U ratio* membrane saturated: unsaturated lipid ratio

Since the membrane consists of lipids and proteins, altering the abundance of FadL and OmpF might affect the total membrane lipid content. The Δ*ompF* strain had a comparable membrane lipid content to MG1655 (Table 3), which indicates that *ompF* deletion did not significantly impact membrane lipid production. However, unlike *ompF*, altering the abundance of *fadL* remarkably affected membrane lipid content. For example, the membrane lipid content of Δ*fadL* is only 62 ± 3 mg/g DCW, which is an 11% decrease compared to MG1655 (P < 0.05). Consistently, Pla-*fadL* had a 13% increase in membrane lipid content relative to MG1655 (P < 0.05) (Table 3). This result indicates that, unlike OmpF, FadL might be involved in membrane lipid synthesis, and therefore altering the abundance of *fadL* affects the membrane lipid content and thus membrane integrity. It should be noted that the relative distribution of the lipid tails is not changed in the Pla-*fadL* strain (Table 3).

## Discussion

Product toxicity is often an obstacle for cost-effective production of biofuels and chemicals [9, 10]. Therefore, construction of robust production organisms tolerant to these biorenewables is critical for industrial applications and has attracted increasing attention in recent years [12, 45, 49, 50]. Given its importance to overall cell function, membrane integrity has become an attractive engineering target for enhancing robustness [13, 24]. In the case of fatty acids, a variety of engineering efforts have been applied to increasing membrane integrity, with mixed results. Most of these engineering strategies focused on altering the distribution of the membrane lipids of *E. coli*, such as by altering the average lipids length or degree of saturation [23, 24], though there have also been efforts to identify an efflux system that can improve fatty acid production [26].

**Fig. 4:**
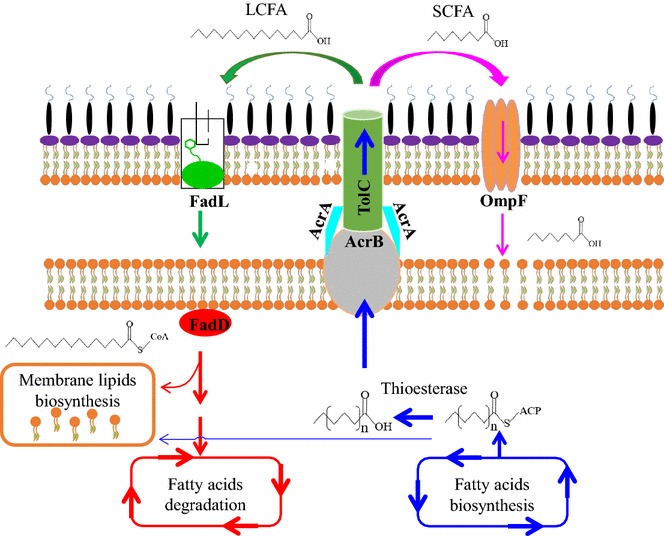
Schematic of the proposed role of *ompF* and *fadL* in maintenance of membrane integrity during fatty acid production in *E. coli*. The elongated acyl-ACP formed during the fatty acids biosynthesis will have two major destinations. Partial acyl-ACPs are hydrolyzed by thioesterase to release free fatty acids. Residual acyl-ACPs serve as precursor for membrane lipids biosynthesis. Among the produced free fatty acids, LCFA (C14–C16) predominates while there is still some SCFA (<C10). It is proposed that LCFA and SCFA are both transported from the cytoplasm directly to the extracellular medium with the AcrAB-TolC complex [26]. However, the low abundance of these compounds in the periplasmic space relative to the extracellular medium results in a driving force for SCFA entry via OmpF and LCFA entry via FadL. LCFAs imported by FadL can be catalyzed by FadD to acyl-CoA, which then serve as fatty acyl precursors for synthesis of phospholipids or enter the β-oxidation cycle for degradation. SCFAs that enter the cell through OmpF, can damage the inner membrane. Increased expression of *fadL* contributes to import of exogenous LCFA, providing precursors for membrane lipids biosynthesis, thereby increasing membrane integrity and supporting fatty acids production, while deletion of OmpF prevents re-entry of the harmful SCFA. LCFA, long chain fatty acids; SCFA, short-chain fatty acids

Here we focused on two membrane proteins, OmpF and FadL, and found that they have distinct effects on maintaining membrane integrity during fatty acid challenge and production. OmpF has been reported to function as the general diffusion porin of *E. coli*, through which a variety of inhibitory molecules, e.g. antibiotics, colicin and SCFA, can enter the cell [32, 51, 52]. Rodriguez-Moya et al. showed that OmpF facilitates transport of C8 into *E. coli*, disrupting intracellular pH and oxidative balance [32]. It has also been suggested that OmpF is involved in the removal of phenylpropanoids from the cell interior [33]. In this study, we further characterized the role of OmpF in maintaining membrane integrity and used the *ompF* deletion strategy to increase fatty acid production. Although we employed the thioesterase specific for release of LCFA (C14–C16), some SCFAs were produced (e.g. C8 and C10) (Additional file 1: Figure S1). These endogenously produced SCFAs can be exported, i.e. by AcrAB-TolC [26], to the extracellular environment. Conversely, they can also re-enter across the outer membrane through *E. coli* porins (e.g. OmpF) (Fig. 4), which can cause severe membrane damage to *E. coli* even at low concentrations [14].

One possible explanation for our observations is that after the endogenously produced fatty acids exit the cell, presumably via AcrAB-TolC [26], some of the SCFA re-enter the cell via OmpF. Deletion of *ompF* blocks this re-entry and thereby increases membrane integrity, which in turn reduces the leakage of important cellular molecules such as Mg^2+^ [14, 53], thereby elevating fatty acid tolerance and production (Fig. 4). The unexpected driving force for such transport may be due to the nature of the AcrAB-TolC transporter. Specifically, this transporter spans the periplasmic space [54–56] and thus the periplasm should be relatively depleted in fatty acids.

Our results demonstrate that, in addition to membrane engineering strategies that alter the distribution of the membrane lipid tails, altering the abundance of membrane protein OmpF can also affect membrane integrity and production of fatty acids, which provides another strategy for future membrane engineering. Increasing the expression of an efflux pump has been shown to improve the production of inhibitory products, such as valine [25] and limonene [8] and these efflux pumps are also an important part of antibiotic resistance [57]. To the best of our knowledge, this is the first demonstration that deletion of a transporter is associated with increased production of a membrane-damaging compound.

In contrast to the *ompF* deletion strategy, deletion of *fadL* was found to decrease membrane integrity, tolerance and production of fatty acid. FadL is the only known outer membrane protein capable of importing exogenous hydrophobic LCFA compounds in *E. coli* [32, 34, 58, 59]. Imported LCFA can be degraded through the β-oxidation pathway as sources of carbon and energy, or serve as precursors for membrane phospholipid biosynthesis [30, 59–61]. Since there was still residual glucose at the end of our experiments (data not shown), it is not likely that the decreased fatty acid tolerance and decreased fatty acid production of the Δ*fadL* mutant was caused by carbon or energy limitations. Membrane lipid biosynthesis in *E. coli* requires acyl chains (C16:0, C16:1 and C18:1), of which there are two sources: (1) endogenous long chain acyl-ACP produced by the fatty acid biosynthesis pathway; and (2) long chain acyl-CoA derived from exogenous LCFA [62, 63]. Upon inactivation of FadL, uptake of exogenous LCFA will be decreased and thus membrane lipid biosynthesis will be impaired (Fig. 4). Our experimental results verify this hypothesis, as membrane lipid content was decreased in the Δ*fadL* strain and increased in the Pla-*fadL* strain. Since lipids are the primary structural component of the membrane, changing the membrane lipid content is likely to alter the membrane integrity. This altered membrane lipid content by Δ*fadL* or Pla-*fadL* does not change the distribution of the different membrane lipid types (Table 3), which suggests that FadL is only responsible for supplying LCFA precursors instead of directly participating in the biosynthesis of phospholipids.

As with OmpF, a driving force for fatty acid uptake via FadL is not expected to exist during fatty acid production. Here, we again refer to the nature of the AcrAB-TolC efflux pump as a possible reason for the existence of this driving force. Since the AcrAB-TolC system spans the periplasmic space [54–56], the periplasm may be depleted of fatty acids relative to the extracellular medium. This direct relationship between *fadL* expression and tolerance of membrane-damaging compounds has been noted elsewhere, specifically in regards to phenylpropanoids [33]. This protective effect of FadL against rutin, naringenin and resveratrol was attributed to FadL’s role in repairing membrane damage, though there is no apparent exogenous source of the fatty acids used for this membrane repair [33].

Current membrane engineering strategies focus on altering membrane lipids composition, such as with the goal of increasing membrane lipid length or S/U ratio, to increase membrane integrity. Our results show that increasing the whole membrane lipid content possibly also contributes to increased membrane integrity, tolerance and production of fatty acids, which may serve as a novel strategy for membrane engineering in the future.

Our qRT-PCR results showed that there is a positive relationship between *fadL* mRNA abundance and fatty acid titer, and they also show that the native *fadL* gene is maintained at a high expression level, which indicates the importance of FadL in maintaining normal phospholipids biosynthesis. Concurrent deletion of *ompF* and increased expression of *fadL* synergistically increased fatty acid tolerance and production, accompanied by increased membrane integrity, possibly due to an increase in membrane lipid content and prevention of re-entry of the SCFA.

Bae et al. [34] found that deletion of *fadD* and overexpression of *fadL* in *E. coli* increased hydroxy long-chain fatty acid production. In that study, it was concluded that overexpression of *fadL* contributes to the improvement in the production of ω-hydroxy palmitic acid, primarily due to increased ability to transport exogenously fed palmitic acid (C16). The present work mainly focuses on the effect of *fadL* overexpression on the import of exogenous LCFA for membrane lipid synthesis and thus maintaining membrane integrity during the production of or challenge with membrane-damaging fatty acids. Prior research showed that deletion of *ompF* or *fadL* in *E. coli* did not affect fatty acid production [26], which is different from our results. There are two possible reasons for this difference: (A) the use of different thioesterases; and (B) the use of different growth conditions. The previous studies used a C8–C14-producing thioesterase enzyme from *U. californica*, while here we used a C14–C16-producing thioesterase from *R. communis*. This previous study also used nutrient-rich LB with 0.4% (v/v) glycerol at 37 °C, while we used the nutrient-poor minimal MOPS with 2% (wt/v) dextrose at 30 °C. It is interesting to note that the studies that identified a positive relationship between OmpF abundance, FadL abundance and phenylpropanoid tolerance were also performed at 30 °C [33]. The use of glycerol in the previous fatty acid production studies may also be a complicating factor. The increase in hydroxy-palmitic acid production upon overexpression of FadL was smaller in the presence of glycerol relative to glucose [34] and the presence of glycerol has previously been reported to alter the phospholipid composition of microbial cell membranes [64–66]. Under different growth conditions, the membrane composition and associated amount of membrane damage caused by the fatty acids is expected to vary, and therefore the roles of OmpF and FadL may differ.

This engineering method appears to increase fatty acid production as a direct function of increased abundance of the microbial biocatalyst. Thus, it differs from a previously described membrane engineering method that increased fatty acid titers by 50% without impacting the final culture OD [23] and evolutionary strain development that improved fatty acid production fivefold while only increasing growth during fatty acid production threefold [50]. The strategy described here also differs from provision of valine-producing *E. coli* with a valine exporter, which increased valine titers by 50% without changing the final OD [25]. Thus, additional strain engineering would be needed in order for this strategy to be effective in improving fatty acid production in fed-batch or continuous culture systems. However, this work clearly demonstrates that these two membrane proteins are two viable engineering targets for improving fatty acid production.

## Conclusions

Membrane damage of the microbial biocatalyst is a widespread problem in the problem of biorenewable fuels and chemicals. Here we have demonstrated two strategies for dealing with membrane damage in our condition. The first is to increase the abundance of FadL, which we propose increases the ability of the organism to repair the membrane damage incurred by fatty acids. The second method is to delete OmpF, which we propose prevents re-entry of the inhibitory product.

## Additional file

**Additional file 1: Figure S1.** Fatty acids profile of *E. coli* MG1655 harboring pXZ18Z plasmid which carries thioesterase gene from *Ricinus communis* and *fabZ* gene from *E. coli*. Some short chain fatty acids (e.g. butanedioic acid, octanoic acid and decanoic acid) were found in the fermentation broth.

## Abbreviations

OmpF: outer membrane porin F
FadL: long-chain fatty acid outer membrane porin
SCFA: short-chain fatty acids
LCFA: long chain fatty acids
C7: heptanoic acid
C8: octanoic acid
C11: undecanoic acid
C14: tetradecanoic acid
C15: pentadecanoic acid
C16:1: palmitoleic acid
C16:0: hexadecanoic acid
C17: heptadecanoic acid
TE: pXZ18Z
DCW: dry cell weight
IPTG: isopropyl-β-d-thiogalactopyranoside
ORF: open reading frame
*ldhA*: lactate dehydrogenase gene
