# Supplementary material for: Improving Escherichia coli membrane integrity and fatty acid production by expression tuning of FadL and OmpF
Source: Microb Cell Fact. 2017 Feb 28;16:38. doi: 10.1186/s12934-017-0650-8 (PMC5331629; doi:10.1186/s12934-017-0650-8)
Supplement: Supplementary file 1 — Additional file 1: Figure S1. Fatty acids profile of E. coli MG1655 harboring pXZ18Z plasmid which carries thioesterase gene from Ricinus communis and fabZ gene from E. coli. Some short chain fatty acids (e.g. butanedioic acid, octanoic acid and decanoic acid) were found in the fermentation broth. [file 12934_2017_650_MOESM1_ESM.docx]

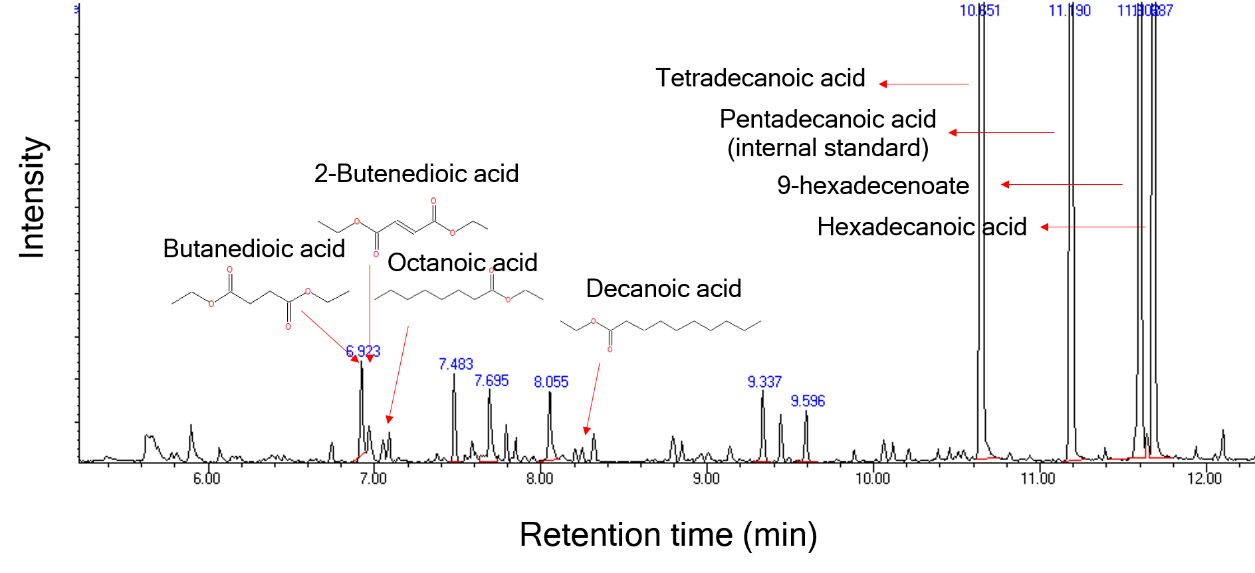


Fig. S1. Fatty acids profile of *E. coli* MG1655 harboring pXZ18Z plasmid which carries thioesterase gene from *Ricinus communis* and *fabZ* gene from *E. coli*. Some short chain fatty acids (e.g. butanedioic acid, octanoic acid and decanoic acid) were found in the fermentation broth.
